# Floating Spleen and Its Relation to Obstetrics and Gynecology

**Published:** 1914-02

**Authors:** 


Floating Spleen and Its Relation to Obstetrics and Gynecology. Montuoro *Ztschr. f. Geburts, und Gynae,* says that floating spleen appears to be a disease peculiar to the feminine sex, as shown by the overwhelming majority of the observed cases. The reason for this has not been satisfactorily explained. Enlargement of the organ alone will not account for it, for while malarial splenic tumors are more frequent in males than in females, prolapse of such a spleen is never found in them. According to a number of authors the spleen becomes congested and enlarged during pregnancy, and this may have some bearing upon the etiology. In general, the factors concerned in the causation of floating spleen comprise enlargements of the organ, changes of the peritoneal folds, reduction of abdominal pressure, relaxation of the abdominal walls, etc., in connection with a traumatic factor or vomiting. While any of these conditions may be present in pregnancy, they do not suffice for an explanation, since otherwise floating spleen would be much more frequent during this period than at other times. Even the changes in the thoracic and abdominal organs caused by the wearing of a corset cannot be considered of etiological significance, because the majority of splenectomies have been performed in peasant women who do not wear corsets. Floating spleen has been observed in all regions of the abdomen and may contract adhesions with any of the abdominal or pelvic organs. Its presence only becomes serious when a twisting of the pedicle takes place because of the severe resulting changes in the splenic structures as well as the inflammatory reaction in the peritoneum and neighboring viscera. If pregnancy occurs in a subject of floating spleen the organ may be compressed between the uterus and pelvis or its pedicle may become twisted, requiring resort to splenectomy. Fortunately, this operation gives a favorable prognosis both as regards the mother and the continuation of the pregnancy. Floating spleen is rarely correctly diagnosed, the tumor being very likely confounded with inflammatory swellings or neoplasms.

